# The genetic basis of resistance and matching-allele interactions of a host-parasite system: The *Daphnia magna-Pasteuria ramosa* model

**DOI:** 10.1371/journal.pgen.1006596

**Published:** 2017-02-21

**Authors:** Gilberto Bento, Jarkko Routtu, Peter D. Fields, Yann Bourgeois, Louis Du Pasquier, Dieter Ebert

**Affiliations:** Basel University, Zoological Institute, Vesalgasse 1, Basel, Switzerland; Fred Hutchinson Cancer Research Center, UNITED STATES

## Abstract

Negative frequency-dependent selection (NFDS) is an evolutionary mechanism suggested to govern host-parasite coevolution and the maintenance of genetic diversity at host resistance loci, such as the vertebrate MHC and R-genes in plants. Matching-allele interactions of hosts and parasites that prevent the emergence of host and parasite genotypes that are universally resistant and infective are a genetic mechanism predicted to underpin NFDS. The underlying genetics of matching-allele interactions are unknown even in host-parasite systems with empirical support for coevolution by NFDS, as is the case for the planktonic crustacean *Daphnia magna* and the bacterial pathogen *Pasteuria ramosa*. We fine-map one locus associated with *D*. *magna* resistance to *P*. *ramosa* and genetically characterize two haplotypes of the *Pasteuria* resistance (PR-) locus using *de novo* genome and transcriptome sequencing. Sequence comparison of PR-locus haplotypes finds dramatic structural polymorphisms between PR-locus haplotypes including a large portion of each haplotype being composed of non-homologous sequences resulting in haplotypes differing in size by 66 kb. The high divergence of PR-locus haplotypes suggest a history of multiple, diverse and repeated instances of structural mutation events and restricted recombination. Annotation of the haplotypes reveals striking differences in gene content. In particular, a group of glycosyltransferase genes that is present in the susceptible but absent in the resistant haplotype. Moreover, in natural populations, we find that the PR-locus polymorphism is associated with variation in resistance to different *P*. *ramosa* genotypes, pointing to the PR-locus polymorphism as being responsible for the matching-allele interactions that have been previously described for this system. Our results conclusively identify a genetic basis for the matching-allele interaction observed in a coevolving host-parasite system and provide a first insight into its molecular basis.

## Author summary

Negative frequency-dependent selection, whereby common genotypes are disfavored, resulting in cyclic change of gene frequencies and maintenance of genetic diversity in host and parasite populations, is one the mechanisms predicted to drive host-parasite coevolution. Specific matching-allele interactions between hosts and parasites are a mechanism predicted to underpin this mode of selection. In spite of in depth research, little is known about the genetic basis of such matching-allele interactions and few empirical examples have been described. Recent research has suggested that the *Daphnia*-*Pasteuria* host-parasite system follows a model of negative frequency-dependent selection. We map a *Daphnia magna* locus of resistance to *Pasteuria ramosa*. We use next-generation genome and transcriptome sequencing to characterize resistant and susceptible haplotypes of the resistance locus. We find large-scale structural polymorphism between resistance locus haplotypes and we find evidence that gene conversion, segment duplication and restricted homologous recombination contribute to produce the observed polymorphisms. We analyse natural populations and find that the resistance locus structural polymorphisms reproduce the matching-allele interactions predicted for the *Daphnia-Pasteuria* system. This work presents rare and conclusive evidence of the genetic basis of matching-allele interactions in host-parasite systems while opening research avenues to find the underlying molecular mechanisms.

## Introduction

Host-parasite interactions are ubiquitous among all living organisms and are thought to represent one of the strongest contributing factors to shaping the evolution of biological organisms [1]. The antagonistic nature of host-parasite interactions leads to reciprocal selection of the antagonists on each other that can drive rapid coevolutionary change [1–3]. Hosts are expected to evolve mechanisms to reduce the likelihood of infection and to minimize the fitness costs associated with infections, while parasites are expected to evolve mechanisms to evade the hosts’ defense mechanisms. Host-parasite interactions are thought to contribute to diversification, speciation, maintenance of sexual reproduction, and maintenance of genetic diversity in natural populations [1, 4–6]. Multiple evolutionary mechanisms have been proposed to underlie host-parasite evolutionary dynamics. These include heterozygote advantage, selective sweeps, and negative frequency-dependent selection (NFDS) [2, 7–9]. NFDS, whereby common host genotypes have a selective disadvantage, can result in balancing selection and is therefore proposed to contribute to the maintenance of genetic diversity in natural populations. The selective disadvantage for common host genotypes comes about because parasites are expected to adapt to these common genotypes [10, 11]. Signatures of balancing selection have been found in gene families associated with disease resistance in vertebrates (the Major Histocompatibility Complex, MHC) and plants (R-gene) [12, 13]. An assumption underlying this form of coevolution is that no parasite can infect all host types and no host can resist all parasite types. The matching-allele-model is one of the genetic mechanisms suggested to prevent the rise of such super-genotypes and thus contributing to the maintenance of genetic diversity [10, 11, 14]. However, despite of in-depth knowledge of the molecular structure of immune-related loci, the genetics underlying the interactions between hosts and parasites have not yet been resolved [15–17].

The *Daphnia–Pasteuria* system is a model for studies in host-parasite coevolution. *Pasteuria ramosa* is an obligate bacterial pathogen of the crustacean *Daphnia magna* that causes strong disease phenotypes with major fitness consequences for the host [8]. In short, feeding hosts pick up dormant *P*. *ramosa* spores. Contact with the host results in the activation of spores, which then attach to the hosts’ foregut. If attachment is successful, the spores penetrate into the *D*. *magna* body cavity initiating infection and disease. *P*. *ramosa* eventually kills the host and its spores are then released into the environment [18]. Importantly, spore attachment is genetically determined and fully consistent with infection success, i.e. resistant host genotypes prevent spore attachment whereas attachment is successful in susceptible host genotypes [19–22]. Here we use the terms resistance and susceptibility to refer to both spore attachment and overall infection.

In this host-parasite system fluctuating selection in natural populations have been observed [23] and the *D*. *magna—P*. *ramosa* interactions follow a matching-allele model with no universally resistant host genotype being found [20–22, 24]. Thus, the *Daphnia*-*Pasteuria* host-parasite system fulfils the core assumptions of models for coevolution by NFDS [10, 11, 14], making it a promising model to explore the underlying genetic mechanisms of host-parasite interactions.

We aimed to investigate the molecular genetic basis of this host-pathogen system and to gain insight into the genetic basis of coevolution by NFDS. Using a Quantitative Trait Locus (QTL) approach on a *D*. *magna* F2 recombinant panel, one large effect QTL associated with resistance to infection by the *P*. *ramosa* C19 genotype was detected [25]. The F2 recombinant panel showed Mendelian segregation of approximately 75% resistant and 25% susceptible genotypes. We build upon this work to explore and characterize the *Pasteuria* Resistance locus (PR-locus) in *D*. *magna*. We show that the PR-locus is highly polymorphic with striking structural genetic polymorphisms and, additionally, gene content and gene expression divergence in the PR-locus between resistant and susceptible haplotypes. The most striking aspect of these differences in gene content is related to a cluster of glycosyltransferase genes located within the PR-locus. Finally, we show that genetic variation at the PR-locus explains variation in resistance to spore attachment observed in natural *D*. *magna* populations following the predictions of a matching-allele model.

## Results

### Fine mapping of *Pasteuria* resistance QTL

**Fig 1:**
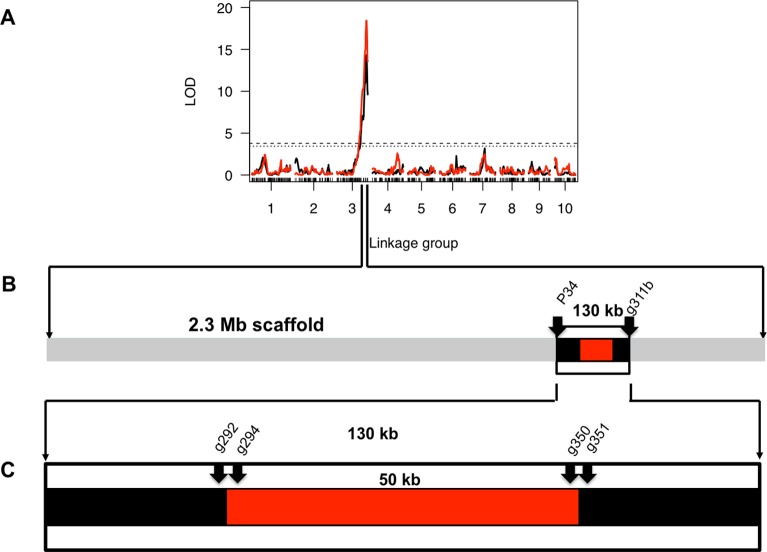
Fine-mapping of the *Pasteuria* resistance locus. A) Quantitative Trait Locus (QTL) analysis of *Daphnia magna* resistance to infection by *Pasteuria ramosa* C19 clone. One large-effect QTL found that explains 59% of variation [25]. B) Break-point mapping of *D*. *magna* PR-locus. Recombination breakpoints analysis determined that the resistance locus is located between markers P34 and g311b. This reduced PR-locus to 136 kb. C) Region within the PR-locus with presumed structural genetic variation–NHR. Markers g292 and g351 are the closest that can be amplified in both parental genotypes of the QTL panel (Xinb3 and Iinb1).

Routtu and Ebert (2015) detected one major effect QTL underlying *D*. *magna* resistance to infection by the *P*. *ramosa* C19 genotype located within a scaffold of approx. 2.3 Mb of the *D*. *magna* draft genome 2.4 (Fig 1A)[25]. We reduced the interval of the *D*. *magna* resistance locus and fine-mapped the QTL interval using microsatellites and SNP markers to find recombination breakpoints within the QTL interval (S1 File). Microsatellite marker P34 and SNP g311b (S1 Table) defined the closest recombination breakpoints at positions 1369860 and 1506194 of scaffold00944 in the *D*. *magna* genome draft 2.4, leaving a mapping interval of approximately 130 kb that we call here the PR-locus (Fig 1B). Within this region no further recombination event was detectable among 360 F2 clones. Interestingly, we detected a genomic region of approximately 50 kb within the interval map where none of the designed genetic markers (g294 and g350) could be amplified in the resistant parental *D*. *magna* clone Iinb1, while the genetic markers placed outside this region (g292 and g351) did amplify in both parent clones (Fig 1C). As genetic markers were designed to match the *D*. *magna* Xinb3 based draft genome (*D*. *magna* 2.4), this result could be explained by structural polymorphism—a single indel polymorphism where the entire 50 kb region is absent in *D*. *magna* Iinb1 genotype or by a genomic region of such high sequence divergence between haplotypes that all the primer pairs based on *D*. *magna* Xinb3 clone would not produce an amplicon with *D*. *magna* Iinb1 DNA.

### PR-locus haplotype sequencing

**Fig 2:**
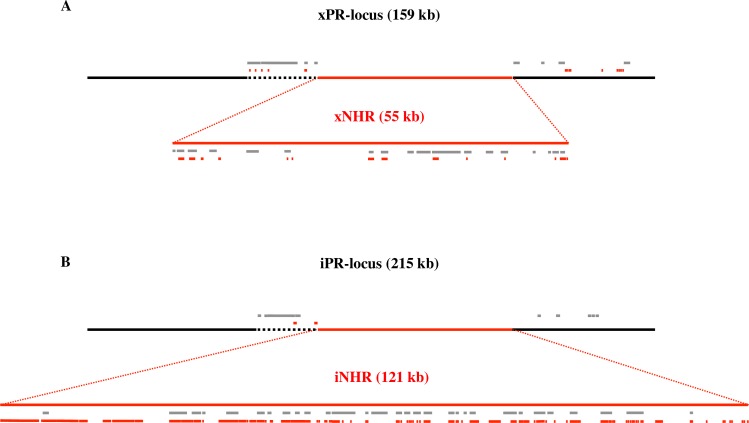
Schematic representation of polymorphism between Xinb3 and Iinb1 PR-locus haplotypes. A) Haplotype xPR-locus. B) Haplotype iPR-locus. iPR-locus is considerably longer than xPR-locus (215 kb to 159 kb). Most of this difference can be explained by differences in the centrally located NHR (121 kb to 55 kb), where little homology between Xinb3 and Iinb1 haplotypes can be found (red line) (expanded for detail). The remaining PR-locus sequence is homologous between the haplotypes (black line). A short region left of NHR is largely made of extra-locus repeats (black dashed line). Extra-locus repeats (grey bars) and intra-locus repeats (red bars) are concentrated in and around the NHR (See expansion for detail).

In order to understand the polymorphism between the parental genotypes we applied high-throughput sequencing and long-read PacBio sequencing of both parental clones with the goal to improve the existing assembly of PR-locus in the *D*. *magna* Xinb3 clone and to obtain an independent *de novo* assembly of the same region in the Iinb1 clone. We obtained two complete haplotypes from the *D*. *magna* clones Xinb3 and Iinb1 for the PR-locus that correspond to the interval between positions 1366653 and 1520041 of the scaffold00944 in draft genome 2.4 and call them the xPR-locus and iPR-locus, respectively. The most striking feature found was that each haplotype contains a large genomic region where little homology was found corresponding to the region where we had previously found amplicon presence/absence polymorphism (Fig 1C). We call this the Non-Homologous Region (NHR), and the haplotypes we obtained from clones Xinb3 and Iinb1 are called xNHR and iNHR, respectively (Fig 2).

### Structural polymorphism in the PR-locus

**Fig 3:**
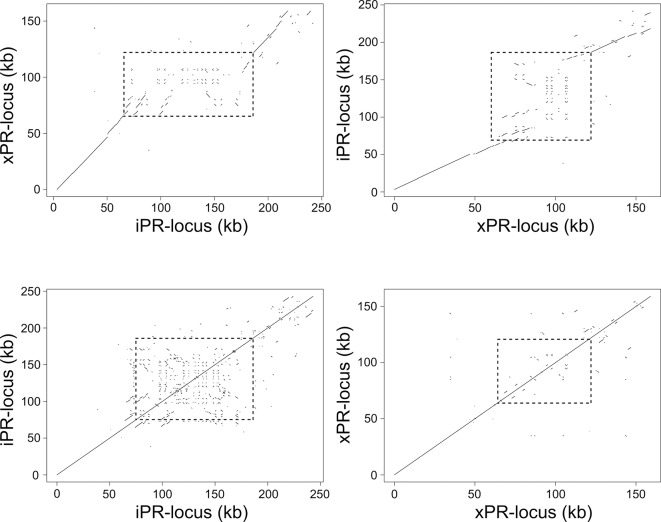
Alignment of *Daphnia magna* Xinb3 and Iinb1 PR-locus haplotypes to itself and to the other. A) Alignment of xPR-locus haplotype to iPR-locus haplotype. B) Alignment of iPR-locus haplotype to xPR-locus haplotype. Reciprocal alignments between PR-locus haplotypes show that at the center (indicated by dashed boxes) is a genomic region with little homology between the haplotypes, whereas at the flanking regions homology between the haplotypes is continuous. This non-homologous region defines the NHR. C) Alignment of iPR-locus to itself. D) Alignment of xPR-locus haplotype to itself. Alignments of each PR-locus haplotype to self reveal that the iPR-locus haplotype has a higher number to intra-locus repeats and that these are repeated more often than in xPR-locus. Intra-locus repeats are concentrated in the NHR.

xPR-locus and iPR-locus differ in their nucleotide lengths: xPR-locus is 159 kb long while iPR-locus is 215 kb long. In addition, considering the entire PR-locus haplotypes 34% of xPR-locus and 46% of iPR-locus have no homology to each other (Fig 2) (S2 Table). However, these differences in length and lack of homology are unevenly distributed across PR-locus. It is the NHR that differs substantially in length: iNHR (from the Iinb1 clone) was 121 kb in length, in contrast to xNHR (Xinb3 clone) with only 55 kb (Figs 2 and 3). The two NHR haplotypes contain only few fragments with homologous sequences: in iNHR a total of 25 kb had a significant alignment in xNHR, representing only 20% of the total sequence; in xNHR only 13.7 kb could be homologized to iNHR (Figs 2 and 3)(S2 Table). This region of non-homology at the NHR contrasts to high homology (>90%) at the flanking regions of the NHR, i.e. in the remainder of the PR-locus (Figs 2 and 3)(S2 Table).

A large proportion of both PR-locus haplotypes was composed of repeated sequences. We divide the repeated sequences in two groups according to the location of their copies: sequences that are repeated in the host genome but outside PR-locus–extra-locus repeats; and sequences that were repeated within PR-locus–intra-locus repeats. A large proportion of both PR-locus haplotypes sequences were made of extra-locus repeats. In spite of the differences observed in length between xPR-locus and iPR-locus haplotypes, both had approx. 25% of their total sequence composed of these extra-locus repeats representing 54.7 kb and 39.9 kb, respectively (Fig 2)(S3 Table). Looking into the distribution of extra-locus repeats we observed that they were unevenly distributed as the NHR contains by far the largest proportion of these extra-locus repeat elements, representing 33% of iNHR and 38% of xNHR (Fig 2)(S3 Table). In addition, the remaining extra-locus repeats found outside the NHR were concentrated in a 20 kb region immediately upstream of NHR (Fig 2)(S3 Table). Interestingly, extra-locus repeats accounted for a significant proportion of sequences non-homologous between PR-locus haplotypes. Specifically, 53% of the non-homologous iPR-locus sequences and 51% of the non-homologous xPR-locus are extra-locus repeats. Second, iPR-locus and xPR-locus diverged in number and nature of intra-locus repeats. In xPR-locus, we detected 14 intra-locus repeats, covering 17.3 kb or 11% of the sequence total (Fig 2)(S4 Table). In contrast, in iPR-locus haplotype we detected 30 intra-locus repeats, representing 68 kb and nearly 32% of the total sequence (Fig 2)(S4 Table). Most of these intra-locus repeats were located within the NHR, specifically 97% and 67% of the intra-locus repeat sequence in iNHR and xNHR, respectively (Fig 2)(S4 Table).

In summary, PR-locus is characterized by dramatic structural polymorphism that in its overwhelming majority is contained within a defined genomic region, the NHR. In particular a large proportion of PR-locus sequences here investigated are non-homologous between the resistant and susceptible haplotypes; a large proportion of both PR-locus haplotypes was composed of repeat elements; the repeat sequences could be repeated extra-locus, intra-locus or both; a large part of the sequence that was non-homologous between the PR-locus haplotypes was composed of extra-locus and/or intra-locus repeats; PR-locus haplotypes diverged in their sequence nucleotide length and in the number and nature of both extra and intra-locus repeats (Figs 2 and 3). The NHR, where most of the variation described here is found, is therefore a strong candidate to harbor variation underlying *D*. *magna* resistance to *P*. *ramosa*.

### Gene annotation in the PR-locus

We annotated the expressed genes in each PR-locus haplotype. Orsini *et al*. (2016) produced an RNAseq database for *D*. *magna* Xinb3 and Iinb1 clones investigated in this article, as well as for *D*. *magna* F1 lineage resulting from a cross between *D*. *magna* Xinb3 and Iinb1 clones [26]. This *D*. *magna* (Xinb3 x Iinb1) F1 clone was in turn used to generate the F2 recombinant panel genotypes used for QTL mapping [27]. In addition to control conditions, the Orsini *et al*. (2016) study also investigated gene expression in the same genotypes when exposed to multiple environmental stress factors, including exposure to spores of *P*. *ramosa* [26]. Using this resource we produced a *de novo* transcriptome and carried out reciprocal blasts between this transcript database and the PR-locus haplotype sequences that we generated from *D*. *magna* Xinb3 and Iinb1 genotypes in order to find which expressed transcripts map to each of the PR-locus haplotypes. We annotated a total of 83 expressed genes that map to the PR-locus haplotypes. Of these, 20 mapped exclusively to the iPR-locus and 18 exclusively to the xPR-locus, whereas 45 annotated expressed transcripts mapped to both haplotypes (S5 Table). The 20 annotated genes that mapped only to the iPR-locus represented one sulfoquinovosyltransferase, and 19 uncharacterized proteins (UP) (S5 Table). The 18 annotated genes that mapped only to the xPR-locus represented five fucosyltransferases, one alpha 1,4-glycosyltransferase, one PC-Esterase and 11 UPs (S5 Table). These observations revealed that the differences in gene content between PR-locus haplotypes resulted for the most part from differences in the representation of fucosyltransferases and UPs. Importantly, all the genes that were exclusive of one or another haplotype, mapped entirely to the NHR region at the center of the PR-locus with the exception of one fucosyltransferase mapping to xPR-locus. This result is consistent with the lack of homology between haplotypes at the NHR. Finally, the 45 expressed transcripts that were shared between the PR-locus haplotypes represented four PC-Esterases, two fucosyltransferases, one methyltransferase, one alpha 1,4-glycosyltransferase, one galactosyltransferase, one sestrin, one DNA mismatch-repair protein, one zinc-finger binding domain, one glutamate synthase, one calcipressin, one spermidine synthase, one acyl-CoA Thioesterase and 29 UPs (S5 Table).

### Gene expression differences between resistant and susceptible genotypes

We investigated differences in expression of genes shared between clones Xinb3 (susceptible to *P*. *ramosa* C19) and Iinb1 (resistant to *P*. *ramosa* C19). Among the 45 transcripts resulting in annotated genes that mapped to both PR-locus haplotypes, 20 were differentially expressed between Xinb3 and Iinb1 clones (S6 Table). Using the Xinb3 clone (the chosen clone for the 2.4 *D*. *magna* draft genome) as the focal genotype we identified 11 upregulated and nine downregulated expressed transcripts (S6 Table). The 11 transcripts upregulated in the Xinb3 clone represented one methyltransferase, one fucosyltransferase, one DNA mismatch-repair protein, one PC-esterase and seven UPs (S6 Table). The nine transcripts downregulated in the Xinb3 clone represented one calcipressin, one DNA mismatch-repair protein, one fucosyltransferase, one sestrin and five UPs (S6 Table). In order to narrow down the number of candidate genes in the PR-locus haplotypes, we compared expression of transcripts mapping to the PR-locus haplotypes between the Xinb3 and Iinb1 clones and the hybrid F1 (Xinb3 x Iinb1) clone. The hybrid F1 clone was resistant to the *P*. *ramosa* C19 genotype just as the Iinb1 clone and in contrast to the Xinb3 clone. Thus, we searched for those transcripts that were consistently down- or upregulated in the Xinb3 clone in comparison to both of the Iinb1 and F1 clones, as those represented the best candidates to underlay the variation in resistance to *P*. *ramosa* observed in the previous QTL study [25]. Only one transcript of calcipressin was downregulated in the Xinb3 clone when compared to both of the Iinb1 and F1 clones. In contrast, seven transcripts were upregulated in the Xinb3 clone, including one methyltransferase, one DNA mismatch-repair protein, and five UPs (S6 Table).

In Orsini *et al* (2016), a number of transcripts were differentially expressed between *P*. *ramosa* infected and non-infected individuals of the same genotype (same *D*. *magna* clone) [26]. We investigated these transcripts to find if any of them would map to our interval. Importantly, we found no significant differences in gene expression between controls and *P*. *ramosa* treatments for transcripts mapping to PR-locus (data not shown) (but see McTaggart *et al*. 2015) [28]. Rather, the significant differences in expression were identified when comparing the control treatments of the Xinb3 and Iinb1 clones. This is not surprising given that we are here investigating the host’s first line of defense, while genes expected to be expressed differently are genes whose expression is induced once the parasite succeeds in infecting its host—the second line of defense [18].

### Structural variation in the NHR is associated with natural variation in resistance to the C1 *P*. *ramosa* genotype

**Fig 4:**
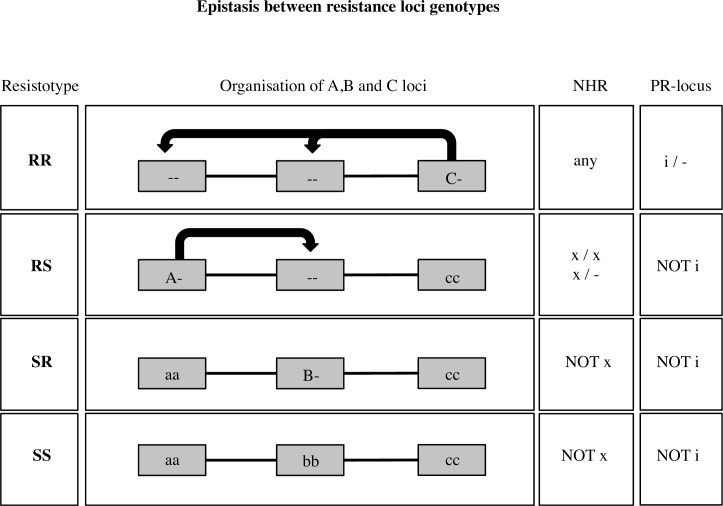
The ABC genetic model for *Daphnia magna* resistance to *Pasteuria ramosa*. *D*. *magna* resistance to *P*. *ramosa* C1 and C19 genotypes was suggested to be controlled by three linked loci (A, B and C) and epistasis between them [22]. Arrows represent dominant epistasis. When dominant allele C is present, the host’s phenotype is RR, irrespectively of the genotypes at loci A and B. Allele C is present within the here described iPR-locus. When the C-locus is homozygote for the recessive allele c, the A-locus is unmasked. With the dominant allele A present at the A-locus, the host’s resistance phenotype is RS irrespectively of genotype at locus B. Allele A is present in the xNHR haplotype. With loci A and C being homozygote for the recessive alleles (cc_aa) and the dominant allele B is present, the host’s phenotype is SR. When all three loci have double recessive genotypes the phenotype is SS. All three loci are located within the here described PR-locus.

**Fig 5:**
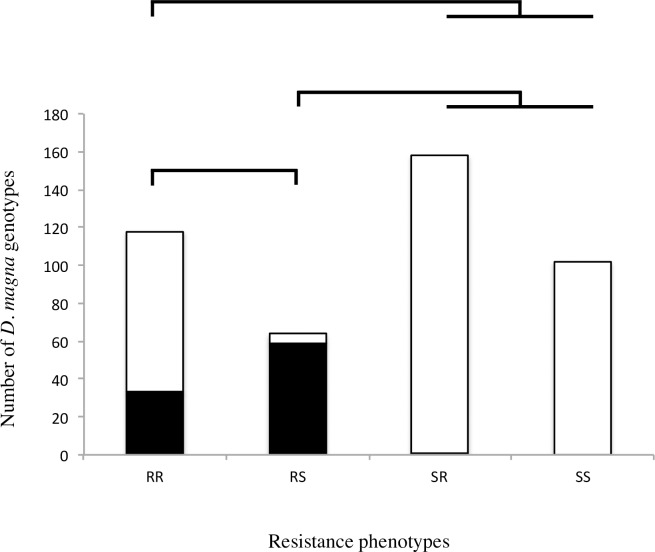
xNHR haplotype association to *Daphnia magna* resistance phenotypes in natural populations. Number of *D*. *magna* genotypes collected in Tvärminne archipelago sorted by resistance phenotype: RR–C1/ C19 double resistant; RS–*P*. *ramosa* C1 resistant and C19 susceptible; SR–*P*. *ramosa* C1 susceptible and C19 resistant; SS—double susceptible. Presence of xNHR haplotype diagnostic markers is denoted in black bars while white denotes absence. Chi-square tests on contingency tables of expected values were applied to the full dataset (P<0.0001) and in pairwise comparisons between phenotypes. Bars indicate comparisons where P-values were significant (*P*<0.0001). xNHR-haplotype presence is associated to RS phenotype whereas absence is associated to SR and SS phenotypes.

One model was suggested, whereby three *D*. *magna* resistance loci govern the *Daphnia*-*Pasteuria* host-pathogen system, regarding the two *P*. *ramosa* genotypes, C1 and C19 [22]. In this model, variation in locus C determines resistance to both *P*. *ramosa* genotypes whereas variation in loci A and B determines *D*. *magna* resistance to *P*. *ramosa* genotypes C1 and C19, respectively. Epistasis between loci can be described as follows: the presence of the resistant allele in C masks the genotypes at loci A and B, and the presence of the resistant allele in A masks the genotype at locus B (Fig 4). A hierarchy of dominance between *D*. *magna* resistance phenotypes is observed: RR (C1, C19 double resistant) > RS (C1 resistant, C19 susceptible) > SR (C1 susceptible, C19 resistant) > SS (double susceptible) [20, 22]. Our analysis so far allows us to conclude that the predicted locus C (Fig 4) is located within PR-locus. However, it does not resolve if different locus C alleles result from structural variation at the NHR or from variation in the flanking region. In addition, since all F2 recombinant clones were either RR (double resistant) or RS (C1 resistant/C19 susceptible) resistance phenotypes, we cannot withdraw any conclusions on whether loci A and B are located within PR-locus even though the three loci are expected to be closely linked [22] (Fig 4). Therefore, we undertook an association study, testing for a link between structural variation at the PR-locus and variation in resistance to *P*. *ramosa* spore attachment in *D*. *magna* clones collected from a metapopulation in the Tvärminne archipelago in Finland. We tested 447 Tvärminne clones from 27 different populations (rock pools) (on average 16.5 clones per population) for resistance to *P*. *ramosa* genotypes C1 and C19 using the attachment test and observed high resistance phenotype diversity between and within the rock pool populations (S7 Table). We then tested two genetic markers (g294 and g350) designed within xNHR unique coding sequences based upon the current draft genome (ver 2.4) for the susceptible *D*. *magna* clone Xinb3 for presence/absence patterns. We had two predictions: i) that these markers (S1 Table) would produce an amplicon when the xNHR haplotype was present either in a homozygote or heterozygote form, but not when it was absent from the tested genotype; and ii) that since the RS phenotype (observed in Xinb3 clone) is dependent on the dominant allele of locus A, these amplicons would be produced irregularly in RR clones, always in RS clones and never in SR (C1 susceptible/C19 resistant) and SS (double susceptible) clones. Our analysis revealed two groups of host genotypes. There were genotypes where the xNHR diagnosis markers amplified together (as does the Xinb3 clone) and other genotypes where none of the markers could be amplified (as is the case for the Iinb1 clone) (Fig 1C). As expected, this amplification pattern was strongly associated to resistance to *P*. *ramosa* C1 genotype. Specifically, clones susceptible to C1 almost never showed xNHR diagnostic marker amplification (resistance phenotypes SR and SS). Clones that are at the same time resistant to C1 genotype and susceptible to C19 genotype (RS) always show amplification (this is also the case for the Xinb3 genotype), whereas double resistant clones (RR) could show amplification or not (Fig 5). The double resistant Iinb1 clone does not show amplification of any of these xNHR diagnostic markers. We tested whether these results would be confirmed within a single *D*. *magna* population. We chose a rock pool population (K-8) with only RS and SR resistance phenotypes being present and predicted that this polymorphism is associated with presence and absence of the xNHR. In our K-8 population sample we found that 56 out of 60 RS clones showed xNHR marker amplification, whereas only one out of 36 SR clones showed such amplification (Table 1). Thus, we find a strong association between presence of xNHR haplotype and RS resistance phenotype, and xNHR absence and C1 susceptibility both within and between populations.

**Table 1:** Association between xNHR haplotype and resistance polymorphism in the K-8 population.

	Host resistance phenotype	
xNHR haplotype	RS	SR
Present	56	1
Absent	4	35

Test for association between the presence of the xNHR and the RS phenotype in the K-8 population. Strong association found between presence/absence of xNHR haplotype and host phenotype (*P<*0.0001).

## Discussion

### NHR structural polymorphisms: evidence of gene conversion and duplication without homologous recombination

The fine mapping and sequence analysis of the *Daphnia magna* PR-locus revealed an unusual pattern of structural polymorphism between haplotypes. Remarkably, we find lack of homology between PR-locus haplotypes in restricted regions of 55 kb and 121 kb, the xNHR and iNHR, respectively (Figs 2 and 3)(S2 Table). In the PR-locus haplotypes, and particularly within the NHR sequences we found a complex pattern of repeated sequences, which likely represent a history of evolutionary events with multiple classes of structural mutations playing a role. The existence of large-scale repetition of sequences found elsewhere in the *D*. *magna* genome, the extra-locus repeats (Fig 2)(S3 Table), argues against horizontal gene transfer in creating the NHR, while suggesting that gene conversion might be a recurrent phenomenon influencing its evolution. The difference in length between the two haplotypes is explained by a far higher prevalence of intra-locus repeats in the iNHR in comparison to the xNHR that suggests a higher number of segment duplication events in iNHR (Fig 2)(S4 Table). Finally, the lack of homology between the two NHR haplotypes together with the observation that this region seem to segregate as one unit in natural populations, suggests the absence of, or very low rates of local recombination.

Taken together, our results indicate that the NHR represents a defined and highly divergent genomic region whose structural genetic variation underlies the natural variation in *D*. *magna* resistance to *P*. *ramosa*.

The characteristics that we find in the NHR of the *D*. *magna* PR-locus largely overlap with what is known of the genetics, origin, structure and evolution of supergenes. Supergenes are clusters of multiple loci, each affecting different traits that together control complex phenotypes within a species and segregate as a block that is characterized by restricted or suppressed recombination [29]. Supergenes can emerge due to new mutations leading to beneficial interactions with closely linked loci, or to structural large-scale mutations such as gene duplication and translocation [29]. Large-scale structural polymorphisms are one of the main reasons for recombination suppression in supergenes and there are examples of supergenes being located in genomic fragments that are absent in alternative haplotypes [29, 30]. Finally, NFDS seems to be the main evolutionary mechanism to maintain supergene polymorphism [29]. Thus, it is tempting to suggest that the NHR of *D*. *magna* PR-locus may represent an immunity supergene.

### Resistance is associated with the NHR in hosts from natural populations

We collected more than 400 clones from a well-studied *D*. *magna* metapopulation located in the Tvärminne archipelago in South-Western Finland and made an association study between their resistance phenotypes for *P*. *ramosa* genotypes C1 and C19 and the presence of diagnostic markers of the xNHR. We find that the presence of the xNHR haplotype is tightly associated to the RS phenotype (C1 resistance and C19 susceptibility), while xNHR markers are absent in *D*. *magna* clones with SR and SS phenotypes (Fig 5). On the other hand, the presence of xNHR markers shows no association with RR phenotypes (Fig 5). We verified the association between xNHR and the RS phenotype in a single population (rock pool K-8), which was polymorphic only for RS and SR phenotypes. In this population the matching-allele matrix–already described for this host-parasite system–is clearly seen [21, 22]. *D*. *magna* clones showing RS phenotype are homozygote or heterozygote for the dominant xNHR, while this haplotype is absent in SR clones (Fig 4) (Table 2). Gene conversion, rare events of homologous recombination at NHR, or errors while determining the resistance phenotypes or the marker could explain the few instances where xNHR diagnostic markers are absent in RS clones or present in SR clones (Table 1)(S7 Table).

**Table 2:** Genotype to phenotype association and hypothesized matching-allele model in the K-8 population.

Host resistance phenotype	Allele model	NHR haplotype
RS	A- BB cc	xNHR
SR	aa BB cc	NOT xNHR

The K-8 population is fully homozygote for the recessive c- allele, thus the absence of RR clones. RS clones have the presence of the dominant A- allele that sits in the xNHR haplotype. SR clones are homozygote for the recessive a- allele as the xNHR haplotype is absent. The absence of SS phenotypes indicates that the population is fixed for the B-allele at the B locus.

Our results are consistent with previous work showing a dominance hierarchy between *D*. *magna* resistance phenotypes and epistasis between resistance loci [20, 22]. The NHR corresponds to the A-locus in these earlier studies. The xNHR contains the dominant allele of the A-locus whereas the iNHR contains the recessive allele. The phenotype associated to xNHR is hidden in RR clones, as its effect is suppressed by the dominant C allele at the C-locus (Fig 4). Conversely, the presence of the xNHR is strongly associated with the RS phenotype and completely absent in SR and SS clones. The presence of the xNHR masks the effect of the B-locus, which defines the SR and SS resistance phenotype polymorphism (Fig 4). In population K-8 the C-locus is apparently fixed for the recessive c-allele, while the B-locus is fixed for the dominant B-allele (Table 2). On the other hand, the results of the QTL mapping leading to PR-locus, is based on a polymorphism at the C-locus (parents are CC—Iinb1, and cc—Xinb3, while the F1 is Cc), because the parental genotypes used, Iinb1 and Xinb3 clones, have RR and RS phenotypes and no other phenotype was found in over 400 tested F2 recombinants [22]. Thus, the C-locus is also located within the PR-locus (Fig 4). Finally, a report of recombination between the three linked resistance loci concluded that the B-locus is located between loci A and C [22], suggesting loci A, B and C loci would all sit within the PR-locus (Fig 4).

Until now few empirical examples of matching-allele interactions have been described in host-parasite systems [31], which can result from this type of genetic interactions being rare. However, in the *D*. *magna*-*P*. *ramosa* system the ease of collecting, large samples are easily available for collection, genotyping and phenotyping. Furthermore, the clonal system of reproduction of *D*. *magna* permits the maintenance of stable genotypes without the need to produce inbred lines [8, 21, 22, 24]. Together, these traits increase the probability of finding existing matching-allele interactions. In addition, many studies of host-parasite systems rely on the overall infection results whereas the infection process requires a series of steps, each with its own genetic basis [18]. In the *D*. *magna*-*P*. *ramosa* system the spore attachment step is the only infection step that fulfils the requirements of a matching-allele model: binary response; lack of environmental variability and; host-parasite genotype-to-genotype interactions. It is possible that by focusing on infection steps that show the same characteristics and using large numbers of host and parasite genotypes, future studies reveal more examples of matching-allele interactions.

### PR-locus gene content polymorphism could underlie natural variation in *D*. *magna* resistance to *P*. *ramosa*

In parallel to large structural polymorphisms found in the NHR region of *D*. *magna* PR-locus we found differences in the gene content between the i- and the x- haplotypes at the PR-locus. Most differences in gene content are associated with genes that map to the NHR region (S4 Table and S5 Table). Gene annotation reveals that genes of the glycosyltransferase family are over-represented within xPR-locus including seven fucosyltransferases, two alpha 1,4-glycosyltransferase and one galactosyltransferase transcripts (S5 Table). In contrast, iPR-locus has only two fucosyltransferase transcripts, one alpha 1,4-glycosyltransferases and one galactosyltransferase (S5 Table). Glycosyltransferases are known to play fundamental roles in innate and acquired immunity-related traits in multiple organisms [32–34]. Thus, the differences in the presence and activity of fucosyltransferases and alpha 1,4-glycosyltransferases indicate that these are good candidates genes that may determine variation in *D*. *magna* resistance to *P*. *ramosa*.

### Future directions–the molecular basis of NFDS

*D*. *magna—P*. *ramosa* is a host-pathogen system where growing evidence suggests NFDS as the primary responsible of the coevolutionary process [20–23]. Here we describe the first steps into the molecular basis of evolution by NFDS and find evidence that suggest a role for glycosyltransferase genes in our study system. Next, it is important to identify which particular genes are responsible for the observed polymorphism. That requires to fine-map the A, B and C loci (Fig 4) and to then carry out functional tests on the remaining candidate genes (e.g. gene knock-outs) to verify their role. Furthermore, it is important to describe more *D*. *magna* PR-locus haplotypes associated with different resistance phenotypes to better understand the extent of the genetic variation associated to *D*. *magna* resistance to *P*. *ramosa* and the relative roles that gene conversion and homologous recombination have in shaping it.

## Methods

### Fine-mapping of *Daphnia magna* resistance QTL

The *D*. *magna* (Xinb3 x Iinb1) F2 recombinant panel is a resource available at the Ebert laboratory in Basel, Switzerland, that was generated from a single cross between the Xinb3 mother clone and the Iinb1 father clone [27]. A QTL analysis based on this resource revealed one major effect QTL for resistance against *P*. *ramosa* genotype C19 [25]. In the region of the major QTL for resistance to *P*. *ramosa*, single nucleotide polymorphism (SNP) and microsatellite markers were designed based on the *D*. *magna* 2.4-genome draft (S1 Table). We amplified each marker via standard PCR and Sanger sequenced them in all F2 clones with a recombination event in the region around the resistance QTL. We then searched for the recombination breakpoints in each F2 recombinant clone.

### Sequencing, assembly and annotation of *Daphnia magna* PR-locus haplotypes

Since the region around the QTL was poorly assembled in version 2.4 of the *D*. *magna* draft genome (http://wfleabase.org/), we undertook a number of additional sequencing and assembly methods in order to better resolve the focal region. For Xinb3 we generated high coverage (~60X) PacBio sequencing in order to perform *de novo* genome assembly. For Iinb1 we took a hybrid Illumina short-read/PacBio long-read approach, generating ~80X 125bp PE Illumina coverage and ~ 15X PacBio long-read coverage (see S1 Methods). We used the *D*. *magna* Xinb3 and Iinb1 haplotype sequences obtained to search for homologies within and between haplotypes and other genomic regions (see S1 Methods). In order to understand how expression of individual genes localized to the focal genome regions and to other parts of the genome differed between the Xinb3 and Iinb1 clones, we conducted a *de novo* transcriptome assembly of the data set described in Orsini *et al*. (2016) (see S1 Methods) [26]. Finally, we constructed a *de novo* annotation of each of the transcripts mapping to PR-locus by performing blastx (nucleotide to protein) searches in the NCBI database (see S1 Methods).

### Haplotype to phenotype association in natural populations

The aim of this assessment was to link the structural polymorphism observed in the QTL panel with genetic variation for resistance in natural populations. *D*. *magna* females were collected from fresh water rock pools in the long term study area of the Tvärminne archipelago, South-Western Finland. The Tvärminne archipelago is composed of many skerry islands of varying sizes, each with multiple rock pools that freeze in winter, forcing the *Daphnia* to survive as sexually produced resting stages called ephippia. It is the location where the ancestor of the *D*. *magna* Xinb3 genotype (our three times selfed reference genome clone) was first collected. Each rock pool represented one population, but together these populations form a metapopulation with frequent migration. Females were freshly hatched from sexually produced resting stages (ephippia) in the wild right after the winter season and thus each of them represented a unique genotype (clone). In the laboratory, we separated females into individual jars initiating a clonal line. Clones were kept in ADaM media at 20°C, fed with *Scenedesmus sp*. three times a week and moved to fresh media once a week [20, 35]. Resistance phenotypes were determined using the attachment protocol described in Duneau *et al*. (2011) [19]. Two cloned *P*. *ramosa* genotypes, C1 and C19, were used in this study [24]. In short, three replicates of each *D*. *magna* clone were placed individually into 96-well plates and exposed for one hour to spores of *P*. *ramosa* C1 or C19 genotypes marked with fluorescein5(6)isothiocyanite [19], after which the attachment of spores to an individual was assessed under fluorescent microscope. Attachment of spores to the esophagus of the host indicated that this host genotype was susceptible to the pathogen genotype tested whereas absence of spore attachment implied host resistance [19]. Primers for genetic structural markers were designed based on the available Xinb3 *D*. *magna* genome draft (version 2.4) at the time. Each primer pair was selected so that it amplified one coding sequence predicted to be present in the annotated genome (S1 Table). Absence or presence of visible amplicons on an agarose gel (1.5% w/v) was used as indicator of PR-locus genotypes (absence indicating homozygotes for absence, while presence indicates homozygotes for presence or heterozygotes). Statistical analysis was based on contingency tables of expected *vs*. observed values to which a Chi-square test was applied to test statistical significance to both the full dataset and to pairwise comparisons between resistance phenotypes.

## Supporting information

S1 File Breakpoint mapping of *D*. *magna* PR-locus. Six replicates were tested for *P*. *ramosa* spore attachment for each F2 recombinant clone. The number of attachment positive tests for each clone is shown. Genotype A represents the Iinb1 resistant clone genotype whereas genotype B represents Xinb3 susceptible clone genotype. Clones and genotypes are colored blue if phenotype or genotype represents dominant resistance, and colored red if phenotype or genotype represents recessive susceptibility. The closest recombination breakpoints found are between markers P34 and g311b. All clones are consistent with the genotype to phenotype association found.
(XLSX)

S1 Methods Supplementary materials and methods and list of supporting literature. (DOCX)

S1 Table Genetic markers. (DOCX)

S2 Table Summary of PR-locus haplotypes genomic organization. (DOCX)

S3 Table Summary of extra-locus repeats distribution. (DOCX)

S4 Table Summary of intra-locus repeats distribution. (DOCX)

S5 Table *D*. *magna* PR-locus annotated genes. Genes that differ in their mapping to the two haplotypes are shown in bold. Genes predicted from the same transcript are shown in the same row. Asterisk denotes genes that are found in two different positions in one haplotype.
(DOCX)

S6 Table Differences of expression in transcripts that map to both PR-locus haplotypes. (+) upregulated; (-) downregulated.
(DOCX)

S7 Table Tvärminne resistance phenotypes across populations (rock pools). (# clones positive for xNHR/# clones).
(DOCX)

## References

[1] Schmid-Hempel P. Evolutionary Parasitology. Oxford University Press; 2011.

[2] Woolhouse MEJ, Webster JP, Domingo E, Charlesworth B, Levin BR. Biological and biomedical implications of the co-evolution of pathogens and their hosts. Nat Genet. 2011; 32(4):569–77. 10.1038/ng1202-569 12457190

[3] Zaman L, Meyer JR, Devangam S, Bryson DM, Lenski RE, Ofria C. Coevolution drives the emergence of complex traits and promotes evolvability. PloS Biol. 2014; 12(12):e1002023 10.1371/journal.pbio.1002023 25514332 PMC4267771

[4] Ebert D, Hamilton WD. Sex against virulence: the coevolution of parasitic diseases. Trends Ecol Evol. 1996 11(2):79–82. 21237766 10.1016/0169-5347(96)81047-0

[5] Salanthé M, Kouyos RD, Bonhoeffer S. The state of affairs in the kingdom of the Red Queen. Trends Ecol Evol. 2008; 23(8):439–45. 10.1016/j.tree.2008.04.010 18597889

[6] Laine AL. Role of coevolution in generating biological diversity: spatially divergent selection trajectories. J Exp Bot. 2009; 60(11): 2957–70. 10.1093/jxb/erp168 19528527

[7] Penn DJ, Damjanovich K, Potts WK. MHC heterozygosity confers a selective advantage against multiple-strain infection. PNAS. 2002; 99(17):11260–4. 10.1073/pnas.162006499 12177415 PMC123244

[8] Ebert D. Host-parasite coevolution: Insights from the *Daphnia*-parasite model system. Curr Opin Microbiol. 2008; 11(3): 290–301. 10.1016/j.mib.2008.05.012 18556238

[9] Wilfer L, Jiggins FM. The dynamics of reciprocal selective sweeps of host resistance and a parasite counter adaptation in *Drosophila*. Evol. 2013; 67(3):761–73. 10.1111/j.1558-5646.2012.01832.x 23461326

[10] Clarke B. The ecological genetics of host-parasite relation-ships In: Taylor AER, Muller R, editors. Genetic aspects of host-parasite relationships. Blackwell, London; 1976 pp. 87–103.

[11] Hamilton W. D. Sex versus non-sex versus parasite. Oikos. 1980; 35:282–290.

[12] Bergelson J, Kreitman M, Stahl EA, Tian D. Evolutionary dynamics of plant R-genes. Science. 2001; 292:2281–5. 10.1126/science.1061337 11423651

[13] Borghans JA, Beltman JB, De Boer RJ. MHC polymorphism under host-pathogen coevolution. Immunogenetics. 2004; 55(11):732–9. 10.1007/s00251-003-0630-5 14722687

[14] Lively CM, Dybdahl MF. Parasite adaptation to locally common host genotypes. Nature. 2000; 405(6787):679–81. 10.1038/35015069 10864323

[15] Tiffin P, Moeller DA. Molecular evolution of plant immune system genes. Trends Genet. 2006; 22(12):662–70. 10.1016/j.tig.2006.09.011 17011664

[16] van Oosterhout C. A new theory of MHC evolution: beyond selection on the immune genes. Proc Biol Sci. 2009; 276(1657):657–65. 10.1098/rspb.2008.1299 18986972 PMC2660941

[17] Ejsmond MJ, Radwan J. Red Queen Processes Drive Positive Selection on Major Histocompatibility Complex (MHC) Genes. PLoS Comput Biol. 2015; 11(11): e1004627 10.1371/journal.pcbi.1004627 26599213 PMC4658181

[18] Ebert D, Duneau D, Hall MD, Luijckx P, Andras JP, Du Pasquier L, Ben-Ami F. A Population Biology Perspective on the Stepwise Infection Process of the Bacterial Pathogen *Pasteuria ramosa* in *Daphnia*. Adv Parasitol. 2016; 91:265–310. 10.1016/bs.apar.2015.10.001 27015951

[19] Duneau D, Luijckx P, Ben-Ami F, Laforsch C, Ebert D. Resolving the infection process reveals striking differences in the contribution of environment, genetics and phylogeny to host-parasite interactions. BMC Biol. 2011; 9:11 10.1186/1741-7007-9-11 21342515 PMC3052238

[20] Luijckx P, Fienberg H, Duneau D, Ebert D. Resistance to a bacterial parasite in the crustacean *Daphnia magna* shows Mendelian segregation with dominance. Heredity. 2012; 108(5):547–51. 10.1038/hdy.2011.122 22167056 PMC3330695

[21] Luijckx P, Fienberg H, Duneau D, Ebert D. A matching-allele model explains host resistance to parasites. Curr Biol. 2013; 23(12):1085–8. 10.1016/j.cub.2013.04.064 23707426

[22] Metzger CM, Luijckx P, Bento G, Mariadassou M, Ebert D. The Red Queen lives: Epistasis between linked resistance loci. Evolution. 2016; 70(2):480–7. 10.1111/evo.12854 26763092

[23] Decaestecker E, Gaba S, Raeymaekers JAM, Stoks R, Van Kerckhoven L, Ebert D, De Meester L. Host-parasite “Red Queen” dynamics archived in pond sediment. Nature. 2007; 450(7171):870–3. 10.1038/nature06291 18004303

[24] Luijckx P, Ben-Ami F, Mouton L, Du Pasquier L, Ebert D. Cloning of the unculturable parasite *Pasteuria ramosa* and its *Daphnia* host reveals extreme genotype-genotype interactions. Ecol Lett. 2011; 14(2):125–31. 10.1111/j.1461-0248.2010.01561.x 21091597

[25] Routtu J, Ebert D. Genetic architecture of resistance in *Daphnia* hosts against two species of host-specific parasite. Heredity. 2015; 114(2): 241–8. 10.1038/hdy.2014.97 25335558 PMC4815634

[26] Orsini L, Gilbert D, Podicheti R, Jansen M, Brown JB, Solari OS, et al *Daphnia magna* transcriptome by RNA-seq across 12 environmental stressors. Sci Data. 2016 1 31 4:170006. 10.1038/sdata.2017.6 PMC5283058 28140384

[27] Routtu J, Hall MD, Albere B, Beisel C, Bergeron RD, Chaturvedi A, et al An SNP-based second-generation genetic map of *Daphnia magna* and its application to QTL analysis of phenotypic traits. BMC Genomics. 2014; 15:1033 10.1186/1471-2164-15-1033 25431334 PMC4301878

[28] McTaggart SJ, Cézard T, Garbutt JS, Wilson PJ, Little TJ. Transcriptome profiling during a natural host-parasite interaction. BMC Genomics. 2015; 16(1):643. 26311167 10.1186/s12864-015-1838-0 PMC4551569

[29] Schwander T, Liebbrecht R, Keller L. Supergenes and complex phenotypes. Curr. Biol. 2014 27(7):R288–94. 10.1016/j.cub.2014.01.056 24698381

[30] Ozias-Akin P, van Dijk PJ. Mendelian genetics of apomixis in plants. Annu. Rev. Genet. 2007 41:509–37. 10.1146/annurev.genet.40.110405.090511 18076331

[31] Thrall PH, Barrett LG, Dodds PN, Burdon JJ. Epidemiological and evolutionary outcomes in Gene-for-Gene and Matching Allele models. Front Plant Sci. 2016 6:1084 10.3389/fpls.2015.01084 26779200 PMC4703789

[32] Thomas CM, Dixon MS, Parniske M, Golstein C, Jones JDG. Genetic and molecular analysis of tomato *Cf* genes for resistance to *Cladosporium fulvum*. Philos. Trans. R Soc Lond B Biol Sci. 1998; 353(1374):1423–24. 10.1098/rstb.1998.0296 PMC1692346 9800204

[33] Becker DJ, Lowe JB. Fucose: biosynthesis and biological function in mammals. Glycobiol. 2003; 13(7):41R–53R. 10.1093/glycob/cwg054 12651883

[34] Ryan SO, Cobb BA. Roles of major histocompatibility complex glycosylation in immune function. Semin. Immunopathol. 2012; 34(3): 425–41. 10.1007/s00281-012-0309-9 22461020 PMC3884644

[35] Ebert D, Zschokke-Rohringer CD, Carius HJ. Within- and between-population variation for resistance of *Daphnia magna* to the bacterial endoparasite *Pasteuria ramosa*. Proc. Biol. Soc. 1998; 265(1410):2127–34.

