# Supplementary material for: The genetic basis of resistance and matching-allele interactions of a host-parasite system: The Daphnia magna-Pasteuria ramosa model
Source: PLoS Genet. 2017 Feb 21;13(2):e1006596. doi: 10.1371/journal.pgen.1006596 (PMC5340410; doi:10.1371/journal.pgen.1006596)
Supplement: S1 Methods — (DOCX) [file pgen.1006596.s002.docx]

**S1 Methods**

**Supplementary methods**

*DNA extraction, sequencing and assembly*

We generated *D. magna* Xinb3 and Iinb1 genomic DNA using a Phenol-Chloroform DNA extraction protocol recommended by PacBio Guidelines for Successful SMRTbell Libraries, adapted by replacing vortexing steps for 2 minute mixing of sample by inversion. DNA for Illumina PE sequencing was extracted using a Gentra Puregene Tissue Kit (QIAGEN), with the inclusion of glycogen in the isopropanol precipitation step. Illumina PE library preparation was done using a Kapa library kit and sequencing on a HiSeq 2500 was done at the ETH Zurich, D-BSSE, Quantitative Genomics Facility in Basel. Assembly of the Xinb3 PacBio sequencing data was done using FALCON v0.3.0. Assembly of the mixture of Illumina 125bp PE and PacBio long-read sequence data derived from the Iinb1 individual was done using DBG2OLC (Ye et al. 2014). Alignment of the focal region was done using LASTZ (Harris 2007). Due to previous inbreeding, we believe the *D. magna* Xinb3 and Iinb1 clones are homozygotes at the region of the resistance QTL.

*Search for sequence homologies*

We used the *D. magna* Xinb3 and Iinb1 haplotype sequences obtained as described above to search for homologies within and between haplotypes and other genomic regions. For that we used 2400 bp segments of each haplotype and used NCBI program BLASTN 2.2.30+. We considered that sequences were homologous when the e-value obtained was bellow 1e^-20^ (Pearson 2013).

*de novo D. magna transcriptome assembly*

Removal of Illumina adapters was made with the use of Trimmomatic (Bolger et al. 2014). Read quality was subsequently assessed through the use of FastQC (Patel & Jain 2012). FASTX toolkit (Pearson et al. 1997) was used to remove reads with Q scores < 30. FastQC was used once again to verify the removal of low-quality reads. Digital normalization of the reads prior to transcriptome assembly was made using the khmer package (Brown et al. 2013; Crusoe et al. 2015). Assembly of the full read data set was made using Trinity v2.1.1 (Grabherr *et al.* 2011; Henschel *et al.* 2012; Haas *et al.* 2013). Transcriptome assessment and removal of low quality transcripts was done using TRANSRATE (Smith-Unna *et al.* 2015). Expression quantification and comparison was made of high-quality transcripts using kallisto (Bray *et al.* 2016; Pimentel *et al*. 2016) and the Rv.3.2 (R core Team 2015) package sleuth (Bray *et al.* 2016; Pimentel *et al*. 2016).

*Transcript mapping and annotation*

We used BLASTN to find transcripts derived from *de novo* RNAseq based transcriptome assembly mapping to each haplotype. Specifically, we made a BLAST search where we queried each assembled haplotype to the full transcript database and extracted all transcripts to which there was a hit with an e-value lower than 1e^-100^. Then, we performed reverse BLAST search of each of these transcripts to the *D. magna* Xinb3 and Iinb1 haplotype sequences and to *D. magna* 2.4-genome draft (derived from *D. magna* Xinb3 genotype). Those transcripts whose best hits were within the haplotype interval were considered to map to the PR-locus haplotype. We then used ExPASy web portal (<http://web.expasy.org/translate/>) to identify Open Reading Frames (ORFs) in each previously selected transcript. For each transcript we selected the largest ORF, or all the ORFs larger than 50 amino acids. Transcripts with no ORF with at least 20 amino acids were removed from the annotation. Finally, we used NCBI blastp search (protein to protein) to find domains contained in each selected ORF. When no domain was found we classified the protein product as Uncharacterized Protein (UP).

**Supplementary references**

Bolger AM, Lohse M, Usadel B. 2014. Trimmomatic: a flexible trimmer of Illumina sequence data. Bioinformatics. 30(15):2114-20.

Bray NL, Pimentel H, Melsted P, Pachter L. 2016. Near-optimal probabilistic RNA-seq quantification. Nat. Biotechnol. 34 (5): 525–7

Brown CT, Scott C, Crusoe MR, et al. (2013) khmer-protocols 0.8.4 documentation. [http://dx.doi.org/10.6084/m9.figshare.878460](http://dx.doi.org/10.6084/m9.figshare.878460)

Crusoe MR, Alameldin HF, Awad S, et al. 2015. The khmer software package: enabling efficient nucleotide sequence analysis. F1000Res. 4:900.

Grabherr MG, Haas BJ, Yassour M, Levin JZ, Thompson DA, Amit I, Adiconis X, Fan L, Raychowdhury R, Zeng Q, *et al*. 2011. Full-length transcriptome assembly from RNA-seq data without a reference genome. Nat Biotechnol. 29(7):644-52.

Haas BJ, Papanicolaou A, Yassour M, Grabherr M, Blood PD, Bowden J, Couger MB, Eccles D, Li B, Lieber M, et al. 2013. De novo transcript sequence reconstruction from RNA-seq using the Trinity platform for reference generation and analysis. Nat. Protocols. 8(8):1494-1512.

Harris RS. 2007. Improved pairwise alignment of genomic DNA. Ph.D. Thesis, The Pennsylvania State University.

Henschel R, Lieber M, Wu L, Nista, PM, Haas BJ, LeDuc R. 2012. Trinity RNA-Seq assembler performance optimization. XSEDE: Bridging from the eXtreme to the campus and beyond. Proceedings of the 1st Conference of the Extreme Science and Engineering Discovery Environment.

Patel RK, Jain M. 2012. NGS QC Toolkit: A Toolkit for Quality Control of Next Generation Sequencing Data. PLoS One. 7(2):e30619.

Pearson WR, Wood T, Zhang Z, Miller W. 1997. Comparison of DNA sequences with protein sequences. Genomics. 46(1):24-36.

Pearson WR. 2013. An Introduction to Sequence Similarity (“Homology”) Searching. Curr. Protocols Bioinformatics. [3: 10.1002/0471250953.bi0301s42.](http://www.ncbi.nlm.nih.gov/entrez/eutils/elink.fcgi?dbfrom=pubmed&retmode=ref&cmd=prlinks&id=23749753)

Pimentel HJ, Bray N, Puente S, Melsted P, Pachter L. 2016. Differential analysis of RNA-Seq incorporating quantification uncertainty. bioRxiv: [http://dx.doi.org/10.1101/058164](http://dx.doi.org/10.1101/058164).

Smith-Unna R, Boursnell C, Patro R, Hibberd J, Kelly S. 2016. TransRate: reference free quality assessment of de novo transcriptome assemblies. Genome Res. pii: gr.196469.115

Ye C, Hill C, Wu S, Ruan J, Ma Z. 2014. DGB20LC: Efficient Assembly of Large Genomes Using the Compressed Overlap Graph. arXiv:1410.2801.
