# Supplementary material for: The genetic basis of resistance and matching-allele interactions of a host-parasite system: The Daphnia magna-Pasteuria ramosa model
Source: PLoS Genet. 2017 Feb 21;13(2):e1006596. doi: 10.1371/journal.pgen.1006596 (PMC5340410; doi:10.1371/journal.pgen.1006596)
Supplement: S1 Table — (DOCX) [file pgen.1006596.s003.docx]

**S1** **Table – Genetic markers**

| **Marker name** | **Forward Primer** | **Reverse Primer** |
| --- | --- | --- |
| P34 | TTCCACTCAGGTCACGACAG | CCAAAAGCGATACCTTCGTT |
| g311b | GCGACCAGACTCTCGGTATT | GACCACCAGCCTCTGAAAAA |
| g292 | ACTGTTTTGGAACGCGAATC | CGCGAAGTGGACTCAAATAA |
| g294 | GCCGATAATTACAAGTTTGTTCT | ATGACGGATCACGATACCAC |
| g350 | TTGGATTGTTGCATCAAGGA | CAGCACCCTGACCAGTTTTT |
| g351 | ACGGACAATCTCAAGCCATT | TTCGTGATGTGTCAGGCAAT |
