# Supplementary material for: The genetic basis of resistance and matching-allele interactions of a host-parasite system: The Daphnia magna-Pasteuria ramosa model
Source: PLoS Genet. 2017 Feb 21;13(2):e1006596. doi: 10.1371/journal.pgen.1006596 (PMC5340410; doi:10.1371/journal.pgen.1006596)
Supplement: S2 Table — (DOCX) [file pgen.1006596.s004.docx]

**S2 Table – Summary of PR-locus haplotypes genomic organization**

| **Haplotype** | **sub-Region** | **Nucleotides (#)** | **Share of haplotype sequence (%)** | **Homologous positions (#)** | **Homologous positions (%)** |
| --- | --- | --- | --- | --- | --- |
| **iPR-locus**  **(Iinb1)** | iNHR | 121265 | 56.40 | 25022 | 20.63 |
|  | Other | 93741 | 43.60 | 91388 | 97.48 |
|  | **Total** | **215006** |  | **116410** | **54.14** |
| **xPR-locus**  **(Xinb3)** | xNHR | 55082 | 34.68 | 11498 | 20.87 |
|  | Other | 103727 | 75.32 | 93963 | 90.59 |
|  | **Total** | **158809** |  | **105461** | **66.41** |
