# Supplementary material for: The genetic basis of resistance and matching-allele interactions of a host-parasite system: The Daphnia magna-Pasteuria ramosa model
Source: PLoS Genet. 2017 Feb 21;13(2):e1006596. doi: 10.1371/journal.pgen.1006596 (PMC5340410; doi:10.1371/journal.pgen.1006596)
Supplement: S3 Table — (DOCX) [file pgen.1006596.s005.docx]

**S3 Table – Summary of extra-locus repeats distribution**

| **Haplotype** | **sub-Region** | **# nucleotides in extra-locus repeats** | **(%) of extra-locus repeats** |
| --- | --- | --- | --- |
| **iR-locus**  **(Iinb1)** | iNHR | 38496 | 31.74 |
|  | pre-NHR | 10173 | 59.84 |
|  | Other | 4472 | 5.83 |
|  | **Total** | **53141** | **24.72** |
| **xR-locus**  **(Xinb3)** | xNHR | 20432 | 37.09 |
|  | pre-NHR | 13226 | 66.13 |
|  | Other | 6080 | 8.24 |
|  | **Total** | **39738** | **25.02** |
