# Supplementary material for: The genetic basis of resistance and matching-allele interactions of a host-parasite system: The Daphnia magna-Pasteuria ramosa model
Source: PLoS Genet. 2017 Feb 21;13(2):e1006596. doi: 10.1371/journal.pgen.1006596 (PMC5340410; doi:10.1371/journal.pgen.1006596)
Supplement: S4 Table — (DOCX) [file pgen.1006596.s006.docx]

**S4 Table – Summary of distribution of intra-locus repeats**

| **Haplotype** | **sub-Region** | **# nucleotides in intra-locus repeats** | **(%) of intra-locus repeats** |
| --- | --- | --- | --- |
| **iR-locus**  **(Iinb1)** | iNHR | 65497 | 54.01 |
|  | pre-NHR | 302 | 1.78 |
|  | Other | 2209 | 2.88 |
|  | **Total** | **68008** | **31.63** |
| **xR-locus**  **(Xinb3)** | xNHR | 10652 | 19.34 |
|  | pre-NHR | 1343 | 6.71 |
|  | Other | 5315 | 6.35 |
|  | **Total** | **17310** | **10.90** |
