# Supplementary material for: The genetic basis of resistance and matching-allele interactions of a host-parasite system: The Daphnia magna-Pasteuria ramosa model
Source: PLoS Genet. 2017 Feb 21;13(2):e1006596. doi: 10.1371/journal.pgen.1006596 (PMC5340410; doi:10.1371/journal.pgen.1006596)
Supplement: S5 Table — Genes that differ in their mapping to the two haplotypes are shown in bold. Genes predicted from the same transcript are shown in the same row. Asterisk denotes genes that are found in two different positions in one haplotype. (DOCX) [file pgen.1006596.s007.docx]

**S5 Table – *D. magna* PR-locus annotated genes.**

Genes that differ in their mapping to the two haplotypes are shown in bold. Genes predicted from the same transcript are shown in the same row. Asterisk denotes genes that are found in two different positions in one haplotype.

| **Transcript ID** | **Gene Annotation (NCBI)** | **Maps to iPR-locus** | **Maps to xPR-locus** |
| --- | --- | --- | --- |
| TRINITY_DN14032_c0_g1_i1 | Zinc-Finger domain | Yes | Yes |
| TRINITY_DN18304_c0_g1_i1 | Glutamate synthase | Yes | Yes |
| TRINITY_DN20075_c1_g1_i2 / TRINITY_DN20075_c1_g1_i1 | Sestrin | Yes | Yes |
| TRINITY_DN12903_c1_g1_i1 | uncharacterized | Yes | Yes |
| TRINITY_DN12903_c2_g1_i1 | uncharacterized | Yes | Yes |
| TRINITY_DN12400_c0_g1_i1 | Calcipressin | Yes | Yes |
| TRINITY_DN15811_c0_g1_i1 | Spermidine synthase | Yes | Yes |
| TRINITY_DN19785_c0_g2_i2 | Fucosyltransferase | Yes | Yes |
| TRINITY_DN19785_c0_g2_i3 | Fucosyltransferase/ uncharacterized | Yes | Yes |
| TRINITY_DN19785_c0_g2_i1 | uncharacterized | Yes | Yes |
| TRINITY_DN19785_c0_g1_i1 | uncharacterized | Yes | Yes |
| TRINITY_DN19785_c0_g3_i1 | uncharacterized | Yes | Yes |
| TRINITY_DN19785_c0_g2_i4 | uncharacterized | Yes | Yes |
| TRINITY_DN17600_c2_g1_i2/ TRINITY_DN17600_c2_g1_i1 | DNA mismatch-repair protein | Yes | Yes |
| TRINITY_DN17600_c1_g1_i1 | uncharacterized | Yes | Yes |
| TRINITY_DN4066_c0_g1_i1 | uncharacterized | Yes | Yes |
| TRINITY_DN8208_c0_g1_i1 | uncharacterized | Yes | Yes |
| TRINITY_DN14941_c0_g2_i1 | uncharacterized | Yes | Yes |
| TRINITY_DN14941_c0_g1_i1 | uncharacterized | Yes | Yes |
| TRINITY_DN14941_c1_g2_i1 | uncharacterized | Yes | Yes |
| TRINITY_DN14941_c1_g1_i1 | uncharacterized | Yes | Yes |
| **TRINITY_DN2983_c1_g1_i1** | **uncharacterized*** | **Yes** | **NO** |
| **TRINITY_DN2983_c0_g1_i1** | **uncharacterized*** | **Yes** | **NO** |
| **TRINITY_DN16752_c1_g1_i1** | **uncharacterized** | **NO** | **YES** |
| **TRINITY_DN16752_c2_g2_i1** | **Alpha 1,4-glycosyltransferase*** | **NO** | **Yes** |
| **TRINITY_DN16752_c2_g3_i1** | **uncharacterized** | **NO** | **Yes** |
| **TRINITY_DN16752_c6_g1_i1** | **uncharacterized** | **NO** | **Yes** |
| **TRINITY_DN16752_c0_g1_i1** | **uncharacterized** | **NO** | **Yes** |
| **TRINITY_DN12789_c0_g1_i1** | **uncharacterized** | **Yes** | **NO** |
| **TRINITY_DN17632_c1_g1_i1** | **uncharacterized*** | **Yes** | **NO** |
| **TRINITY_DN17632_c0_g1_i1** | **uncharacterized** | **Yes** | **NO** |
| **TRINITY_DN17632_c0_g2_i1** | **uncharacterized** | **Yes** | **NO** |
| **TRINITY_DN18257_c0_g1_i3** | **uncharacterized** | **Yes** | **NO** |
| **TRINITY_DN18257_c0_g1_i2** | **uncharacterized** | **Yes** | **NO** |
| **TRINITY_DN18257_c0_g1_i1** | **uncharacterized** | **Yes** | **NO** |
| **TRINITY_DN4507_c0_g1_i1** | **uncharacterized** | **NO** | **Yes** |
| **TRINITY_DN6327_c0_g1_i1** | **uncharacterized*** | **Yes** | **NO** |
| **TRINITY_DN6327_c1_g2_i1** | **uncharacterized*** | **Yes** | **NO** |
| **TRINITY_DN92_c0_g1_i1** | **uncharacterized** | **Yes** | **NO** |
| **TRINITY_DN18917_c1_g1_i1** | **uncharacterized** | **Yes** | **NO** |
| **TRINITY_DN18917_c1_g2_i1** | **uncharacterized** | **Yes** | **NO** |
| **TRINITY_DN18917_c1_g2_i3** | **uncharacterized*** | **Yes** | **NO** |
| **TRINITY_DN18917_c1_g2_i5** | **uncharacterized** | **Yes** | **NO** |
| **TRINITY_DN12789_c0_g1_i1** | **uncharacterized*** | **Yes** | **NO** |
| **TRINITY_DN2843_c0_g2_i1** | **Fucosyltransferase/**  **Fucosyltransferase** | **NO** | **Yes** |
| **TRINITY_DN2843_c0_g1_i1** | **Fucosyltransferase** | **NO** | **Yes** |
| **TRINITY_DN2809_c0_g2_i1** | **Fucosyltransferase** | **NO** | **Yes** |
| **TRINITY_DN2809_c0_g1_i1** | **Fucosyltransferase** | **NO** | **Yes** |
| **TRINITY_DN21286_c0_g1_i1** | **PC-Esterase** | **NO** | **Yes** |
| **TRINITY_DN15336_c3_g3_i2** | **uncharacterized** | **NO** | **Yes** |
| **TRINITY_DN15336_c3_g3_i1** | **uncharacterized** | **NO** | **Yes** |
| **TRINITY_DN15336_c3_g1_i2** | **uncharacterized** | **NO** | **Yes** |
| **TRINITY_DN15336_c3_g1_i1** | **uncharacterized** | **Yes** | **Yes** |
| TRINITY_DN18537_c1_g1_i4 | Acyl-CoA Thioesterase | Yes | Yes |
| **TRINITY_DN18537_c1_g1_i3** | **uncharacterized** | **NO** | **Yes** |
| **TRINITY_DN20768_c0_g1_i1** | **uncharacterized*** | **NO** | **Yes** |
| **TRINITY_DN5412_c0_g2_i1** | **Sulfoquinovosyltransferase** | **Yes** | **NO** |
| **TRINITY_DN5412_c0_g1_i1** | **uncharacterized** | **Yes** | **NO** |
| **TRINITY_DN9602_c0_g1_i1** | **uncharacterized*** | **Yes** | **NO** |
| TRINITY_DN2903_c0_g2_i1/ TRINITY_DN2903_c0_g1_i1 | Methyltransferase | Yes | Yes |
| TRINITY_DN18179_c2_g1_i2 | PC-Esterase | Yes | Yes |
| TRINITY_DN18179_c2_g1_i1 | PC-Esterase | Yes | Yes |
| TRINITY_DN18179_c2_g1_i4 | PC-Esterase | Yes | Yes |
| TRINITY_DN18179_c2_g1_i3 | PC-Esterase | Yes | Yes |
| TRINITY_DN18179_c3_g1_i2 | uncharacterized | Yes | Yes |
| TRINITY_DN18179_c3_g1_i1 | uncharacterized | Yes | Yes |
| TRINITY_DN11011_c0_g2_i1 | uncharacterized | Yes | Yes |
| TRINITY_DN19605_c1_g1_i4 | uncharacterized | Yes | Yes |
| TRINITY_DN19605_c1_g1_i3 | uncharacterized | Yes | Yes |
| TRINITY_DN19605_c1_g1_i1 | Galactosyltransferase | Yes | Yes |
| TRINITY_DN19605_c1_g1_i2 | uncharacterized | Yes | Yes |
| TRINITY_DN15780_c0_g1_i1 | uncharacterized | Yes | Yes |
| TRINITY_DN16606_c1_g1_i1 | uncharacterized | Yes | Yes |
| TRINITY_DN16606_c1_g1_i2 | uncharacterized | Yes | Yes |
| TRINITY_DN16606_c0_g1_i1 | uncharacterized | Yes | Yes |
| TRINITY_DN16606_c2_g1_i1 | Alpha 1,4-Glycosyltransferase/PC-Esterase/ | Yes | Yes |
| **TRINITY_DN18379_c1_g1_i2/ TRINITY_DN18379_c1_g1_i1** | **Fucosyltransferase** | **NO** | **Yes** |
| TRINITY_DN18537_c1_g1_i2 | uncharacterized | Yes | Yes |
| TRINITY_DN8221_c0_g2_i1 | uncharacterized | Yes | Yes |
| TRINITY_DN8221_c0_g1_i1 | uncharacterized | Yes | Yes |
| TRINITY_DN16675_c1_g1_i6 | uncharacterized | Yes | Yes |
| TRINITY_DN16675_c1_g1_i2 | uncharacterized | Yes | Yes |
| TRINITY_DN16675_c1_g1_i1 | uncharacterized | Yes | Yes |
