# Supplementary material for: The genetic basis of resistance and matching-allele interactions of a host-parasite system: The Daphnia magna-Pasteuria ramosa model
Source: PLoS Genet. 2017 Feb 21;13(2):e1006596. doi: 10.1371/journal.pgen.1006596 (PMC5340410; doi:10.1371/journal.pgen.1006596)
Supplement: S6 Table — (+) upregulated; (-) downregulated. (DOCX) [file pgen.1006596.s008.docx]

**S6 Table** – **Differences of expression in transcripts that map to both PR-locus haplotypes**: (+) upregulated and (-) downregulated.

| **Transcript ID**  **(Orsini *et al* 2016)** | **Gene Annotation** | ***D. magna* Iinb1 (res)** | ***D. magna* (Xinb3xIinb1) F1**  **(res)** | ***D. magna* Xinb3**  **(sus)** |
| --- | --- | --- | --- | --- |
| TRINITY_DN12400_c0_g1_i1 | Calcipressin | + | + | - |
| TRINITY_DN14941_c1_g2_i1 | uncharacterized | + |  | - |
| TRINITY_DN16675_c1_g1_i1 | uncharacterized | + |  | - |
| TRINITY_DN17600_c2_g1_i1 | DNA mismatch-repair protein | + | - | - |
| TRINITY_DN18179_c3_g1_i2 | uncharacterized | + |  | - |
| TRINITY_DN18537_c1_g1_i2 | uncharacterized | + |  | - |
| TRINITY_DN19785_c0_g2_i3 | Fucosyltransferase/ uncharacterized | + |  | - |
| TRINITY_DN19785_c0_g2_i4 | uncharacterized | + |  | - |
| TRINITY_DN20075_c1_g1_i1 | Sestrin | + |  | - |
| TRINITY_DN11011_c0_g2_i1 | uncharacterized | - | + | - |
| TRINITY_DN17600_c2_g1_i2 | DNA mismatch repair protein | - | - | + |
| TRINITY_DN15336_c3_g1_i2 | uncharacterized | - | - | + |
| TRINITY_DN16606_c1_g1_i1 | uncharacterized | - |  | + |
| TRINITY_DN16606_c1_g1_i2 | uncharacterized | - | - | + |
| TRINITY_DN16752_c1_g1_i1 | uncharacterized | - | - | + |
| TRINITY_DN18179_c2_g1_i4 | PC-Esterase | - |  | + |
| TRINITY_DN18537_c1_g1_i3 | uncharacterized | - | - | + |
| TRINITY_DN19605_c1_g1_i3 | uncharacterized | - | - | + |
| TRINITY_DN19605_c1_g1_i4 | uncharacterized | - |  | + |
| TRINITY_DN19785_c0_g2_i2 | Fucosyltransferase | - |  | + |
| TRINITY_DN2903_c0_g1_i1 | Methyltransferase | - | - | + |
| TRINITY_DN18304_c0_g1_i1 | Glutamate synthase |  | - | + |
| TRINITY_DN19605_c1_g1_i1 | Galactosyltransferase |  | - | + |
| TRINITY_DN18537_c1_g1_i1 | uncharacterized |  | - | + |
| TRINITY_DN15811_c0_g1_i1 | Spermine synthase |  | - | + |
| TRINITY_DN14032_c0_g1_i1 | Zinc-Finger domain |  | - | + |
| TRINITY_DN16752_c2_g2_i1 | Alpha 1,4-glycosyltransferase |  | - | + |
| TRINITY_DN17600_c1_g1_i1 | uncharacterized |  | - | + |
| TRINITY_DN14941_c0_g1_i1 | uncharacterized |  | - | + |
| TRINITY_DN19132_c0_g2_i1 | uncharacterized |  | - | + |
| TRINITY_DN18179_c3_g1_i1 | uncharacterized |  | - | + |
| TRINITY_DN20075_c1_g1_i2 | Sestrin |  | - | + |
