# Supplementary material for: The genetic basis of resistance and matching-allele interactions of a host-parasite system: The Daphnia magna-Pasteuria ramosa model
Source: PLoS Genet. 2017 Feb 21;13(2):e1006596. doi: 10.1371/journal.pgen.1006596 (PMC5340410; doi:10.1371/journal.pgen.1006596)
Supplement: S7 Table — (# clones positive for xNHR/# clones). (DOCX) [file pgen.1006596.s009.docx]

**S7 Table – Tvärminne resistotypes across populations (rock pools)**.

(# clones positive for xNHR/# clones).

| **Population** | **Resistotype** | | | | **Total** |
| --- | --- | --- | --- | --- | --- |
|  | **RR** | **RS** | **SR** | **SS** |  |
| **3** | 9/11 | 1/1 | 0/1 | 0/7 | 10/20 |
| **4** | 1/1 | 3/3 | 0/15 | 0/1 | 4/20 |
| **5** | 0 | 3/3 | 0/12 | 0/1 | 3/16 |
| **6** | 0 | 8/8 | 0 | 0/2 | 8/10 |
| **7** | 6/10 | 1/1 | 0/2 | 0/7 | 7/20 |
| **8** | 0 | 0 | 0/10 | 0/10 | 0/20 |
| **9** | 0/1 | 11/11 | 0/5 | 0/3 | 11/20 |
| **10** | 0/2 | 1/1 | 0/16 | 0/1 | 1/20 |
| **14** | 0 | 7/7 | 0/3 | 0/7 | 7/17 |
| **17** | 1/3 | 1/1 | 0/16 | 0 | 2/20 |
| **19** | 6/6 | 1/1 | 0/13 | 0 | 7/20 |
| **21** | 0/4 | 4/4 | 0/6 | 0/5 | 4/19 |
| **23** | 0 | 0 | 0/14 | 0/3 | 0/17 |
| **25** | 0/8 | 0 | 0/6 | 0/2 | 0/16 |
| **26** | 0/5 | 3/4 | 0/7 | 0/4 | 3/20 |
| **27** | 0/9 | 0/1 | 0/6 | 0/4 | 0/20 |
| **28** | 0/8 | 4/5 | 0/3 | 0 | 4/16 |
| **31** | 0/9 | 0 | 1/6 | 0/3 | 1/18 |
| **34** | 0/12 | 3/3 | 0/3 | 0/1 | 3/19 |
| **36** | 0/2 | 0/1 | 0/7 | 0/9 | 0/19 |
| **37** | 0/5 | 0 | 0/6 | 0/10 | 0/21 |
| **38** | 1/6 | 8/9 | 0/1 | 0/2 | 9/18 |
| **39** | 0/7 | 0 | 0 | 0/11 | 0/18 |
| **41** | 9/9 | 0 | 0 | 0/9 | 9/18 |
| **42** | 0 | 5/5 | 0 | 0 | 5/5 |
| **Total** | **33/118** | **64/69** | **1/158** | **0/102** | **98/447** |
